# The expression analysis of long noncoding RNAs PCAT-1, PCAT-29, and MER11C in bipolar disorder

**DOI:** 10.1186/s12888-024-05974-y

**Published:** 2024-07-23

**Authors:** Niloofar Dini, Mohammad Taheri, Zeinab Shirvani-Farsani

**Affiliations:** 1 Department of Cell and Molecular Biology, Faculty of Life Sciences and Biotechnology, Shahid Beheshti University, Tehran, Iran; 2 Institute of Human Genetics, Jena University Hospital, Jena, Germany

**Keywords:** lncRNAs, *PCAT-1*, *PCAT-29*, *MER11C*, Bipolar disorder, Biomarkers

## Abstract

Long non-coding RNAs (lncRNAs) are transcripts with a length of usually more than 200 nucleotides (nt) that have promised functions in varied biological processes. lncRNAs participate in the regulation of differentiation, development, and function of the brain. Thus, their dysregulation might play important roles in the etiology of neurological disorders such as BD. In this study, the expression level of PCAT-1, PCAT-29, and MER11C lncRNAs was evaluated in the blood of BD patients compared to the control group. Peripheral blood mononuclear cells of 50 BD type I patients and 50 healthy individuals were isolated. The RNAs were extracted and cDNA was synthesized. Then, the expression level of the desired lncRNAs was measured through Real-Time PCR. The expression levels of PCAT-29 and MER11C lncRNAs were significantly lower in BD patients compared to controls. However, the expression level of PCAT-1 was not significantly different between these two sets of samples. According to the ROC curve, PCAT-29 and MER11C had significant diagnostic power for the differentiation of BD patients from controls. Taken together, our results indicate dysregulation of two lncRNAs in patients with BD and the possible roles of these lncRNAs in the neuropathology of bipolar disorder.

**Supplementary Information:**

The online version contains supplementary material available at 10.1186/s12888-024-05974-y.

## Introduction

Long non-coding RNAs (lncRNAs) are transcripts with a length of usually more than 200 nucleotides (nt). lncRNAs have promised roles in varied biological routes, including RNA processing and editing, dosage compensation, genomic imprinting and development, cell fate determination and others [1, 2]. lncRNAs play an important role in neuronal differentiation, brain development, and neurogenesis [3]. Moreover, the dynamic expression of lncRNAs is a critical regulator of neuronal differentiation and synaptogenesis by controlling BDNF, DBX1, Nrcam, etc. [4]. Dysregulation of lncRNAs is reported in neurological disorders including schizophrenia, autism spectrum disorder (ASD), multiple sclerosis, Parkinson’s, Huntington’s, and Alzheimer’s diseases [5, 6]. Mounting evidence has shown lncRNAs as possible biomarkers for various neurological disorders [6–8]. In addition, several studies have indicated that some lncRNAs such as lincRNA-p21, lincRNA-PINT, SNHG6, and MALAT1 have been altered in bipolar disorder (BD) [9–11]. However, many lncRNAs in the pathobiology of this psychiatric illness have not yet been studied.

In the current study, we investigated the expression levels of three lncRNAs, namely PCAT-1, PCAT-29 and MER11C in peripheral blood mononuclear cells of patients with BD and healthy controls. These lncRNAs were selected based on their participation in the neuropathology of central nervous system disorders.

PCAT1 (Prostate cancer-associated transcript-1), located at chromosome 8q24 is a lncRNA that was originally identified in prostate cancer [12]. Other studies have assessed the role of PCAT1 in neurological diseases. For instance, Seki et al. (2019) reported that the expression level of PCAT1 was enhanced in patients with major depressive disorder (MDD) compared with healthy controls [7]. PCAT1 was upregulated in glioma stem cells (GSC) and induced cell proliferation by reducing the expression of miR-129-5p and increasing the expression of HMGB1 [13]. The reduction of PCAT1 expression was observed in glioblastoma compared to brain cancer stem cells; consequently, dysregulated PCAT1 may be a new therapy in brain tumors [14]. PCAT29 is a tumor suppressor gene that inhibits cell proliferation, migration, and metastasis [15]. PCAT-29 lncRNA upregulation was reported in patients with major depressive disorder [7]. MER11C is a noncoding retroelement gene located on chromosome 11 [16]. The expression of this lncRNA was elevated in patients with MDD compared with healthy controls [7]. lncRNAs are implicated in the regulation of neuronal differentiation, development, and function [3]. Thus, their dysregulation might play important roles in the etiology of neurological disorders such as BD. In the current study, we assessed the expression levels of three lncRNAs in patients with BD compared with controls.

## Materials and methods

In the current study 50 BD type I patients (manic state) who were referred to the Behavioral Science Research Center of Imam Hussein Hospital, Tehran, Iran was enrolled. All patients were examined by the same psychiatrist according to DSM-5 criteria [17]. Exclusion criteria were a history of epilepsy and head trauma, current drug abuse, encephalitis or mental retardation, and any other neurologic disorder. The control group was selected from the same source and consisted of 50 healthy individuals for whom psychiatric disorders were ruled out. Age, sex, and ethnicity were matched between the two groups. Ethical Committee of Shahid Beheshti University of Medical Sciences issued ethical approval for the current study. Informed consent was signed by all participants.

### RNA extraction and cDNA synthesis

5 ml of the patients’ and control groups’ peripheral blood samples were collected in an EDTA tube. Peripheral blood mononuclear cells (PBMC) were isolated using the Ficoll-Paque PLUS standard procedure (GE Healthcare Life Sciences, Piscataway, NJ, USA). RNA extraction was done using the RNX kit (EX6101, Cinnagen, Tehran, Iran) according to the manufacturer’s guidelines. DNA contamination was eliminated using DNaseI (Fermentas, Lithuania). cDNA was synthesized using 3 µg of purified total RNA by Applied Biosystems High-Capacity cDNA Reverse Transcription Kits (PN:4,375,575) in a total volume of 20 µl reaction mixture.

### Quantitative real-time PCR

Quantitative real-time PCR was executed in The Applied Biosystems StepOnePlus (Applied Biosystem, Foster City, CA, USA) using 10 µl of BIOFACT™ 2X Real-Time PCR Master Mix, 10 ng cDNA, and 200 nM of each primer. Each sample was analyzed twice. The lncRNA expression was measured in comparison with B_2_M as a reference gene using related primers (B2M: F-CCACTGAAAAAGATGAGTATGCCT & R- CCAATCCAAATGCGGCATCTTCA, PCAT-1: F-CGCAAAGGAACCTAACTGGAC & R-GTCTCCGCTGCTTTATAACCC, PCAT-29: F-CAGCACCATCACATGCCTCCA & R- CCAAATCAAGTCACATGCCGAT and MER11C: F-AAACTTGCTGATTTTGTGGCTT & R-TGTTGGCTGGTCTGTGAAATA). The mean of ΔCT for both groups was calculated, and eventually, the relative expression of each gene was estimated by ratio, i.e. 2 ^− ΔΔCt^ (fold change) as described by Livak [18].

### Statistical analysis

Statistical analyses were done using GraphPad Prism 8 (GraphPad Software, Inc., San Diego, CA, USA). Kolmogorov–Smirnov test was used for testing the normality of the data. RNA expression levels between different lncRNA groups were compared using Independent Student’s T-test or Mann-Whitney test. The correlation of gene expression with clinical features including age, disease duration, and disease onset was assessed with multiple regression model (Least squares). Data were shown as mean ± standard deviation (SD). P-values < 0.05 were considered significant. In addition, we utilized the Bonferroni correction for obtaining adjusted P values (q values). Bonferroni corrected P value was obtained by the original α-values/the quantities of analysis of the dependent variable. Receiver Operating Characteristic (ROC) curve was executed to evaluate the specificity and sensitivity of RNA expression differences in nominated genes.

## Results

### General demographic information

This study included 50 BD type I patients and 50 healthy individuals as the control group. The controls matched with the patients for age (*p* = 0.68) and gender (In both groups 30% of the patients were female). Demographic details for all patients are shown in Table 1.

**Table 1:** Demographic data of participants

Demographic data	BD patients	Healthy controls
**Number of participants**	50	50
**Number of female/males**	15/35	15/35
**Mean age (years old)**	36.50 ± 9.32	33.62 ± 5.21
**Mean age of disease onset (years)**	32.64 ± 8.04	-
**Mean disease duration (years)**	3.86 ± 2.66	-

### Expression assays

**Fig. 1:**
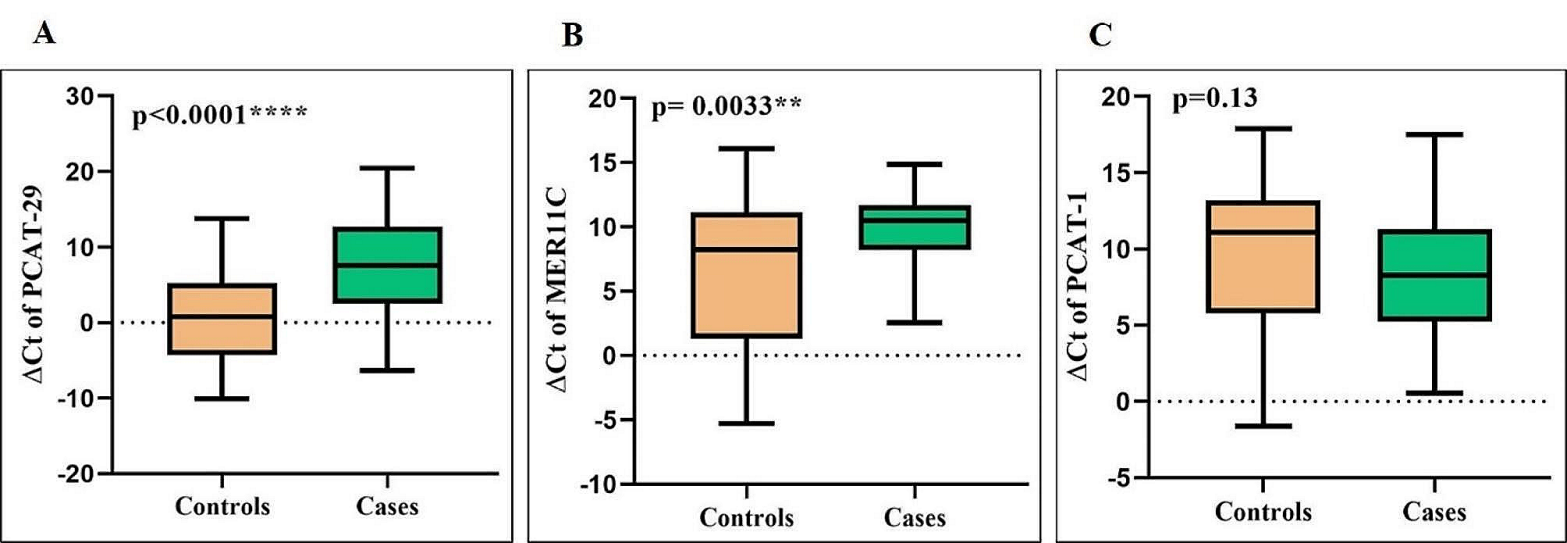
The analysis of expression levels of three lncRNAs in the PBMCs of BD patients and healthy controls. The normalized expression of PCAT-29 (**A**), MER11C (**B**), and PCAT-1 (**C**). (The normalized expression= ∆Ct values = Ct _Target gene_ – Ct _Housekeeping gene_). The Mann-Whitney U test was used to examine the difference in lncRNA expression between the two groups (**P value < 0.01 and ****P value < 0.0001)

We assessed the expression levels of three lncRNAs (PCAT-29, PCAT-1, and MER11C MER11C) between total cases and controls. The expression levels of PCAT-29 (*p* < 0.0001, fold change= -39.5) and MER11C (*p* = 0.0033, fold change= -11.27) were significantly reduced in BD patients (Fig. 1A and B). Moreover, after adjustment, all P values remained significant (q value < 0.0001 for PCAT-29 and q value = 0.0002 for MER11C).

The expression levels of PCAT-29 and MER11C were 39.5 and 11.27 times lower in bipolar patients in comparison to healthy controls, respectively. There was no significant difference in the level of PCAT-1 between cases and controls (*p* = 0.056) (Fig. 1C). The raw data have showed in the supplementary file 1.

**Fig. 2:**
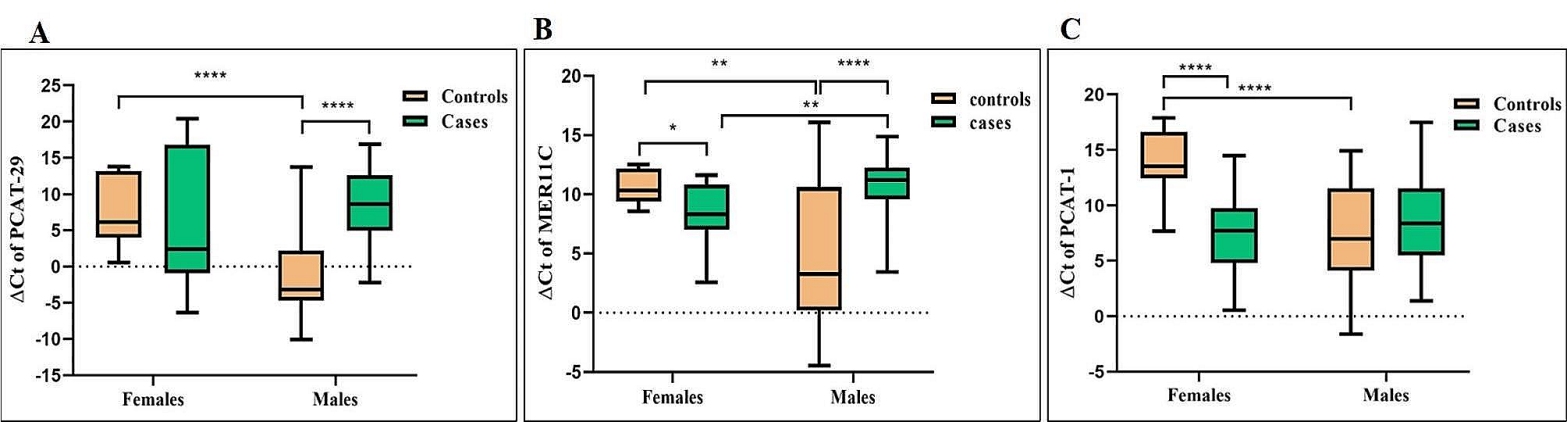
The analysis of expression levels of three lncRNAs in the PBMCs of BD patients (male and female) versus healthy controls (male and female). The normalized expression of PCAT-29 (**A**), MER11C (**B**), and PCAT-1 (**C**). (The normalized expression= ∆Ct values = Ct Target gene – Ct Housekeeping gene). Two-way ANOVA were used to analyze the lncRNA expression levels in subgroups. (*P value < 0.05, **P value < 0.01 and ****P value < 0.0001)

There was a sex-differentiated expression for these lncRNAs. The expression of lncRNA PCAT-29 was significantly decreased in male BD patients compared to male controls (*p* < 0.0001). However, for female patients, it was not significant (*p* = 0.39) (Fig. 2A).

We reported up-regulation of MER11C in female BD patients compared with female controls (*p* = 0.011). For male BD individuals, it was significantly lower than male controls (*p* < 0.0001) (Fig. 2B). Notable, the expression of lncRNA PCAT-1 was significantly higher in female BD patients than in controls women (p​​< 0.0001, 22.31-fold). However, there was no significant difference in the expression of this lncRNA between male BD patients and controls (*p* = 0.42) (Fig. 2C).

We reported up-regulation of PCAT-29 (*p* < 0.0001), PCAT-1 (*p* < 0.0001), and MER11C (*p* = 0.0022) in male controls compared with female controls. Furthermore, expression MER11C was reported to be reduced in male cases compared with female cases (*p* = 0.005). Lastly, the expression of PCAT-29 (*p* = 0.13) and PCAT-1 (*p* = 0.37) was not different between female cases and male cases (Table 2). The raw data have showed in the supplementary file 2.

**Table 2:** The results of the Fold change of three lncRNAs in BD patients compared to healthy controls

		Total patients vs. Controls (50 vs. 50)	Male patients vs. Male Controls (35 vs. 35)	Female patients vs. Female Controls (15 vs. 15)	Female patients vs. Male patients (15 vs. 35)	Female controls vs. Male Controls (15 vs. 35)
PCAT-29	Posterior beta of Fold Change(2^−∆∆Ct^)	-39.5	-48.3	1.1	-1.34	49.1
	Adjusted P Value	< 0.0001****	< 0.0001****	0.39	0.13	< 0.0001****
PCAT-1	Posterior beta of Fold Change(2−∆∆Ct)	2.54	1.3	22	1.2	31.2
	Adjusted P Value	0.056	0.42	< 0.0001****	0.37	< 0.0001****
*MER11C*	Posterior beta of Fold Change(2−∆∆Ct)	-11.27	-21.43	3.78	-4.02	54.7
	Adjusted P Value	0.0002***	***p*** < 0.0001****	0.011*	0.005**	0.0022**

Significant values (*P* < 0.05) indicated in bold. Minus (-) indicates reduced expression.

### Correlation studies

Subsequently, we examined the relationship between gene expression and demographic/clinical information of patients with BD by utilizing the multiple regression model. There was no significant correlation between the expression level of PCAT-29, PCAT-1 & MER11C. Furthermore, no significant correlation was found between PCAT-29, PCAT-1, and MER11C expression with age, disease duration, and disease onset (Table 3).

**Table 3:** Results from the multiple regression model. Parameter estimates of PCAT-29 expression and other variables

Independent Variables	β	SE	t Value	*P* Value
Intercept (PCAT-29)	*-5.59*	*20.75*	*0.26*	*0.78*
MER11C	*1.44*	*1.434*	*1.008*	*0.32*
PCAT-1	*0.93*	*1.526*	*0.61*	*0.54*
Age (year)	*-0.22*	*478*	*4.784e-015*	*> 0.99*
Disease onset (year)	*-0.30*	*488*	*6.306e-015*	*> 0.99*
Disease duration (year)	*0.73*	*481*	*1.540e-014*	*> 0.99*
MER11C * PCAT-1	*-0.00095*	*0.09*	*0.010*	*0.99*
MER11C * Age	*0.0036*	*171*	*2.131e-016*	*> 0.99*
MER11C * Disease onset	*0.0061*	*176*	*3.584e-016*	*> 0.99*
MER11C * Disease duration	*-0.074*	*175*	*4.366e-015*	*> 0.99*
PCAT-1* Age	*-0.0062*	*338*	*1.838e-016*	*> 0.99*
PCAT-1* Disease onset	*-0.0082*	*338*	*2.431e-016*	*> 0.99*
PCAT-1* Disease duration	*0.0101*	*338*	*2.992e-016*	*> 0.99*
Age * Disease onset	*0.0081*	*0.012*	*0.67*	*0.50*
Age * Disease duration	*-0.048*	*0.107*	*0.44*	*0.65*
Disease onset * Disease duration	*0.053*	*0.143*	*0.37*	*0.71*

### Diagnostic potential analysis

**Fig. 3:**
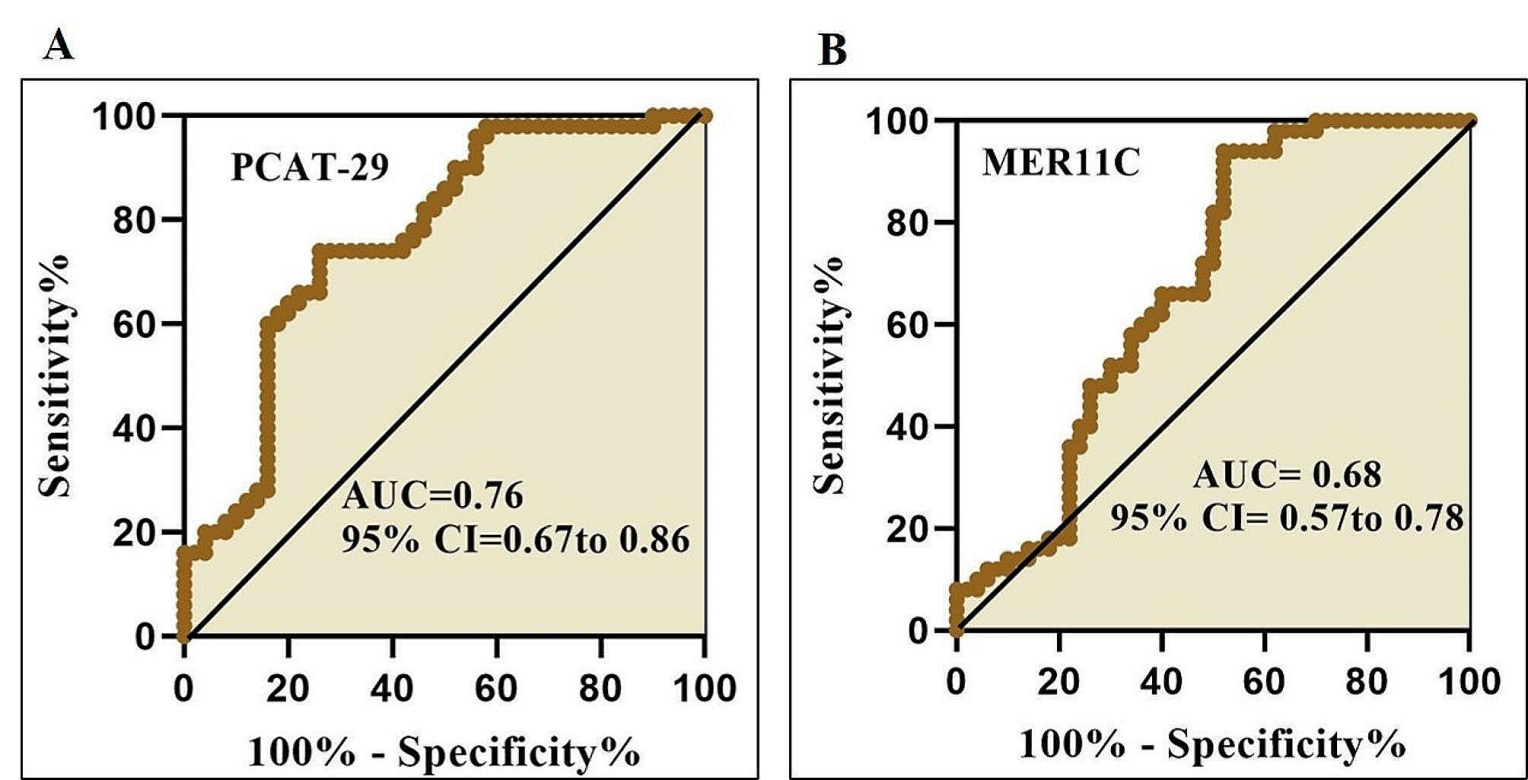
ROC curve analysis of PCAT-29 (**A**) and MER11C lncRNA (**B**) expression for differentiating BD patients from healthy controls. The sensitivity was plotted against the

ROC curve analyses indicated that expression levels of *PCAT-29 and* MER11C could differentiate BD patients and normal healthy with AUC values of 0.76 and 0.68 (Fig. 3; Table 4).

specificity for each threshold value. AUC: area under curve.

**Table 4:** The results of ROC curve analysis for two differentially expressed lncRNAs in patients with bipolar disorder and healthy controls

	AUC ± SD	Sensitivity %	Specificity %	cut off	*P* Value
**PCAT-29**	0.76 ± 0.08	74	74	4.02	< 0.0001
**MER11C**	0.68 ± 0.06	66	60	> 9.4	0.0024

## Discussion

Recently, great importance is paid to long noncoding RNAs in the literature and their role in various disease pathogenesis such as psychiatric disorders, is vastly studied. Several lncRNAs are reported to be expressed in brain neural development [19]. Multiple neurological disorders are nowadays linked to lncRNA dysregulation [5, 20]. Also, they could act as biomarkers for various diseases. We assessed the expression of PCAT-1, PCAT-29, and MER11C lncRNAs in BD patients, proposed to be altered in MDD patients [7]. BD pathogenesis is yet to be known, possible reported pathways include disorder in mitochondrial function, neuronal-glial plasticity, monoaminergic signaling, and inflammatory homeostasis [21].

To the best of our knowledge, this is the first study in the literature to evaluate the expression of these lncRNAs in BD patients. PCAT-1 is an oncogenic lncRNA. It acts as a regulator of cell proliferation, apoptosis, migration, and invasion. In a real-time PCR-based investigation, Seki et al. studied 83 lncRNAs that have been previously linked with the brain. They reported increasing in the lncRNAs MER11C, PCAT1, and PCAT29 expression in patients with MDD compared to healthy controls [7]. While, the findings of our study in BD patients compared to healthy individuals, showed a significant decrease in PCAT-29 and MER11C expression and no significant change in PCAT1 expression. This might indicate that these lncRNAs may have another regulatory mechanism or pathway in the pathoetiology of BD compared to MDD.

PCAT-1 levels are increased with the histone deacetylase inhibitor i.e., suberoylanilide hydroxamic acid (SAHA) [22]. SAHA is stated to have improving effects on depressive-like behaviors in mice [23]. PCAT-29 is another lncRNA, which has played roles such as inhibition of cell proliferation, migration, tumor growth, and metastases [24].

PCAT-29 dysregulation was consistently noticed in BD patients of both sexes compared with sex-matched controls. On the contrary, PCAT-1 expression levels were only different only in female subgroups. Also, dysregulation of MER11C was inconsistent in sex-matched analysis. These inconsistencies of results between sexes could be due to different courses and clinical features of BD in men and women [25]. Importantly, the expression of these three lncRNAs differed between males and females in healthy controls and in BD patients except for PCAT-1. This may indicate possible effects of gender on the expression of these lncRNAs in two case and control groups.

Notably, dysregulation of these lncRNAs is consistent with the hypothesis of inflammatory responses in the pathogenesis of BD [26]. PCAT-29 has anti-tumorigenic effects, its downregulation in BD patients of this study may lead to apoptosis disorder in neurons.

MER11C is one of the human polypyrimidine tract-binding protein-associated splicing factors (hPSFs) binding RNAs. The binding of RNA by hPSF endorses transformation and tumorigenesis by reversing the suppression of proto-oncogene translation by PSF [27].

PTB (Polypyrimidine tract-binding protein) is involved in the alternative splicing of early mRNA (Pre-mRNA) and almost all steps of mRNA expression. PTB plays an important role in the development and differentiation of neurons including neuronal differentiation, transcriptional programs, neurogenesis, and synaptic maturation. PTB is restricted to neuronal progenitor cells, glial cells, and other non-neuronal cells, whereas the nPTB protein is specifically expressed in neurons. During neuronal differentiation, miR-124 targets PTB mRNA to reduce PTB levels and enable the induction of nPTB, thereby inducing neuronal differentiation through effects on splicing and translation [28]. Hence, lncRNA MER11C may have a differentiation role in neurons, by binding to the PTB protein-binding factor, and its reduced expression in BD patients may hinder the growth and differentiation of neurons. More accurate studies with a larger sample size are needed to reveal the exact mechanism of lncRNAs in bipolar disorder.

The results obtained from the ROC showed that the area under the curve (AUC) for PCAT-29 and MER11C was 0.76 and 0.68 respectively. Our results show that MER11C and PCAT-29 could separate MS patients and controls. However, none of these two lncRNAs could solely differentiate BD patients from normal controls with suitable diagnostic values. In addition, we analyzed the correlation of the expression of lncRNAs and age, disease onset, and disease duration (Table 3). Despite the dysregulation of PCAT-29 and MER11C in the blood of BD patients, their expression levels did not show any correlation with age, disease duration, or disease onset. Moreover, there was no significant correlation between the expression of these lncRNAs with each other. This lack of correlation could potentially be due to the small sample size of the study.

Taken together, our study shows dysregulation of two lncRNAs, PCAT-29 and MER11C in BD patients compared to controls. Moreover, our findings may help develop sensitive and specific biomarkers for BD. However, this study has some limitations, including a lack of functional investigations with larger sample sizes, expression assessment in postmortem brain tissues or cerebrospinal fluid, and cellular studies that should be done to elucidate the involvement of lncRNAs in BD pathophysiology.

### Electronic supplementary material

Below is the link to the electronic supplementary material.

Supplementary Material 1

Supplementary Material 2

## Data Availability

The datasets used and/or analyzed during the current study are available from the corresponding author on reasonable request.
